# Psychological factors predict adherence to methotrexate in rheumatoid arthritis; findings from a systematic review of rates, predictors and associations with patient-reported and clinical outcomes

**DOI:** 10.1136/rmdopen-2015-000171

**Published:** 2016-01-20

**Authors:** Holly F Hope, James Bluett, Anne Barton, Kimme L Hyrich, Lis Cordingley, Suzanne M M Verstappen

**Affiliations:** 1 NIHR Manchester Musculoskeletal Biomedical Research Unit, Central Manchester University Hospitals NHS Foundation Trust, Centre for Musculoskeletal Research, Institute of Inflammation and Repair, The University of Manchester, Manchester, UK; 2 Arthritis Research UK Centre for Genetics and Genomics, Centre for Musculoskeletal Research, Institute of Inflammation and Repair, The University of Manchester, Manchester, UK; 3 Arthritis Research UK Centre for Epidemiology, Centre for Musculoskeletal Research, Institute of Inflammation and Repair, The University of Manchester, Manchester, UK

**Keywords:** Rheumatoid Arthritis, Methotrexate, DMARDs (synthetic), Epidemiology

## Abstract

Treatment response to methotrexate (MTX) for rheumatoid arthritis (RA) is not universal and non-adherence may partially explain this. The aims of this systematic review were to: (1) summarise existing rates of adherence to MTX, (2) identify predictors of adherence to MTX, and (3) assess the association between non-adherence and patient outcomes. The authors conducted a systematic search of papers published from January 1980 to February 2015 in PubMed, PsycINFO, EMBASE and CINAHL databases. Studies were eligible for inclusion if: (1) MTX was used as monotherapy or in combination with other therapies, (2) MTX was used in an RA or inflammatory polyarthritis population, (3) adherence was defined and measured as the extent to which patients followed their MTX regimen during the period of prescription, and (4) it was an original piece of research. In total, 10 studies met the inclusion criteria and 8 were evaluated as high quality. Rates of adherence ranged from 59% to 107%, and exposed differences in definitions of adherence, study methodologies and sample heterogeneity. A number of potential predictors of MTX adherence were identified; the strongest being related to beliefs in the necessity and efficacy of MTX, absence of low mood, mild disease and MTX monotherapy. Furthermore, 3 studies tested the association of adherence with disease activity as an outcome measure; all 3 found non-adherence associated with poor treatment response. This systematic review shows the importance of adherence to MTX treatment and summarises the associated modifiable factors.

## Introduction

Methotrexate (MTX) was recommended as the first-line therapy for the management of rheumatoid arthritis (RA) by EULAR in the 2013 guidelines and by the National Institute for Health and Care Excellence (NICE) clinical guidelines published in 2009 and updated again in 2013.^1^ ^2^ The recommendation was based on the evidence that MTX has the best drug retention rate (persistence), and equivocal or superior efficacy, in comparison with other synthetic disease-modifying antirheumatic drugs (sDMARDs).^3^ However, response to MTX is not universal; only 28–45% of patients achieved disease activity score (DAS)-defined remission (DAS28<2.6) 1 year after starting MTX monotherapy.^4^ ^5^ In an observational study, with a longer follow-up, remission was observed to drop to 6% and 14%, at 2 and 5 years, respectively.^6^ Response to MTX therapy is likely to be determined by a number of factors but adherence to the treatment regimen may be important.

Adherence, defined by the WHO as “the extent to which the patient's behaviour—taking medication, following a diet, and/or executing lifestyle changes, corresponds with agreed recommendations from a health-care provider” has long been recognised as an important factor in response to treatment.^7^ In today's society non-adherence to medication contributes to increasing healthcare costs with one study reporting a cost to the National Health Service (NHS) in the UK of £300 million every year due to medicines wastage.^8^ There are a range of behaviours that could constitute non-adherence, ranging from patients who do not take their medication at all (complete non-adherence), drug holidays (a period of time of taking no medication), and catch-up dosing (following a drug holiday, an increased dosing frequency to catch-up on missed doses). Adherence was reported to be highest for acute illnesses and reduced with long-term drug use, with substantial reductions seen beyond 6 months of treatment in chronic conditions such as RA.^9–11^

There have been a few systematic reviews of adherence to DMARDs.^12–17^ A review by Pasma *et al*^12^ identified that sDMARD use in the 6 months prior to antitumour necrosis factor initiation and the belief that taking the medication is necessary increased adherence. However, in the review, pharmacological therapies for RA were grouped together to estimate overall adherence rates and investigate predictors. Since MTX is the sDMARD of first choice, it is imperative to have accurate estimates of adherence rates to MTX in the RA population, the effect this has on clinical response, and to investigate potential modifiers of adherence which may be used as targets for intervention. Early interventions to improve adherence to MTX may reduce the need for more aggressive and expensive therapies in the future.

The aims of this systematic review were therefore to (1) obtain an overview of rates of adherence to MTX reported in the literature; (2) evaluate possible predictors of adherence; and (3) describe the strength of association between adherence to MTX and patient-reported and clinical outcomes in patients with RA.

## Methods

### Search strategy

EMBASE, MEDLINE, CINAHL (Cumulative Index to Nursing and Allied Health Literature) and PsycInfo databases were searched from January 1980, until February 2015, using Patient Intervention Comparison Outcome (PICO) search methodology to build the following strategy.^18^ (P) rheumatoid or arthritis patient population; (I) MTX as an intervention; and (O) adherence as a measured study predictor or outcome. The PICO comparison (C) category was not applicable and dropped from the search design. Synonyms for each PICO category were defined and the database search identified abstracts that included a synonym from each category in the title, original title, abstract, subject heading, name of substance or registry word fields (see online supplementary table S1).

### Study inclusion

Studies obtained from the systematic search were eligible for inclusion if: (1) MTX was used as a monotherapy or in combination with other DMARDs, (2) MTX was used in a RA or inflammatory polyarthritis (IP) population, (3) adherence was defined and measured as the extent to which patients followed their MTX regimen during the period of prescription, and (4) it was an original piece of research.

Titles and abstracts obtained from the search were independently evaluated by two researchers (JB and HFH) for inclusion and, where there was a disagreement, adjudicated by a third reviewer (SMMV). In studies evaluating other therapies in addition to MTX therapy, abstracts were excluded where adherence to the overall regimen, rather than to MTX specifically, was assessed. Where original research published since 2013 met the other inclusion criteria but only existed as an abstract, thesis or conference proceedings, efforts were made to contact the authors to obtain a manuscript, and were excluded if the information required to evaluate the quality of these studies was unavailable. Relevant reviews and opinion articles were retrieved in order to cross-reference to ensure all relevant articles were included. The full papers were obtained for the resulting list and reviewed in a similar fashion to the abstracts of published papers. Papers were included where MTX was prescribed in combination with other drugs, provided adherence to MTX had been calculated separately, papers that provided overall adherence rates only were excluded. If included papers used multiple methods to measure adherence, we describe the methods and report the results specific to MTX adherence.

### Quality assessment

The quality of the included studies was formally assessed using an adapted measure from the systematic review of Pasma *et al*.^12^ The quality assessment consisted of 16 items, premised on the recommendations from Sanderson *et al*^19^ that state observational studies should be evaluated on the use of appropriate methods to: (1) select participants, (2) measure exposure and outcome variables, (3) control confounding, (4) reduce bias, and (5) analyse data. See online supplementary table S2 to review all the items in tool. Papers that scored 7 or more out of 10, or 14 or more out of 17 were considered to be of high quality.

### Evidence synthesis

We assessed the association between possible predictors of adherence and the effect size of the association. This evidence was evaluated with reference to the quality of the study, based on the definition of strong, moderate, weak and conflicting evidence of van Tulder *et al*.^20^

## Results

**Figure 1:**
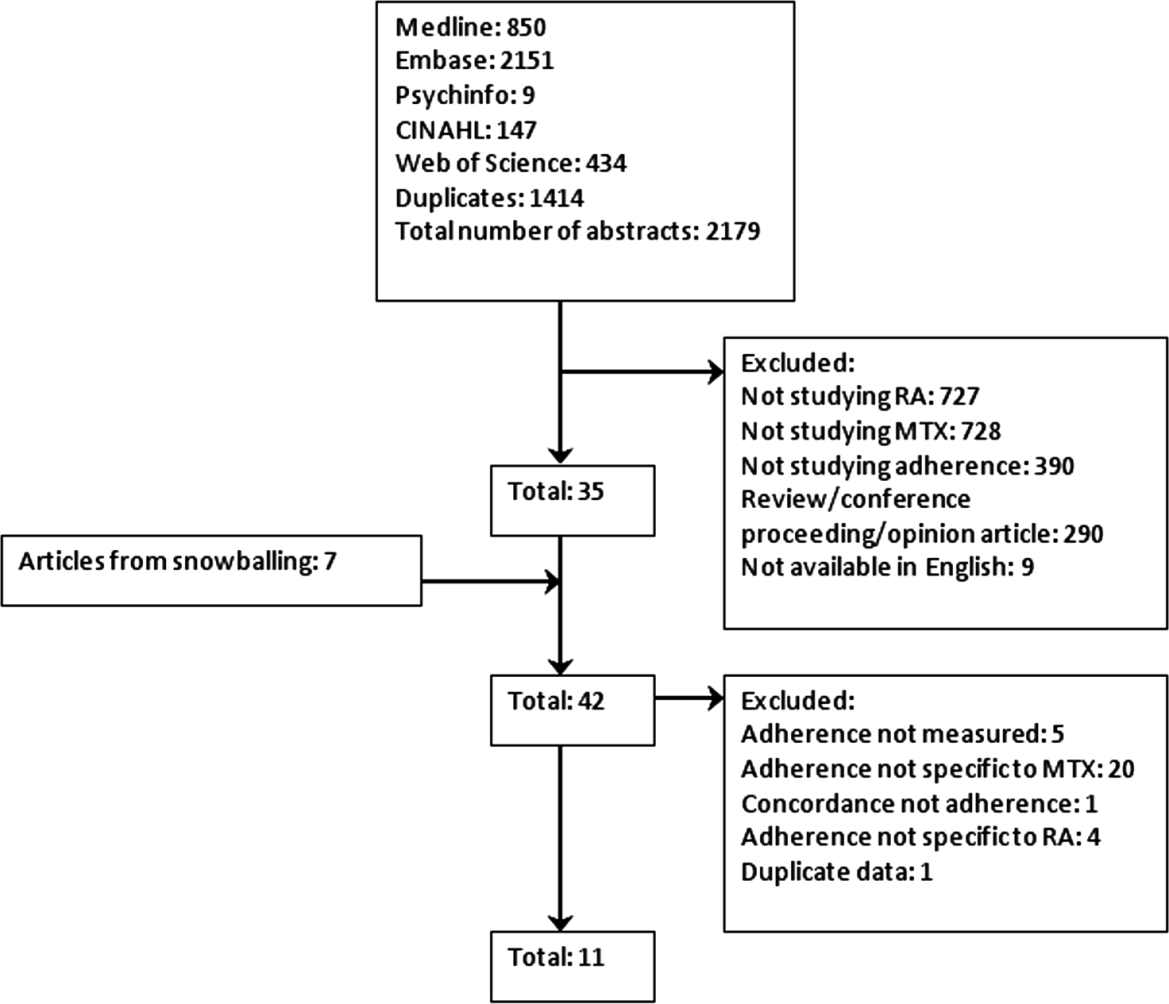
Flow diagram to show the article selection process (MTX, methotrexate; RA, rheumatoid arthritis).

The systematic search generated 1778 abstracts and 27 articles were selected for full paper review, of which 10^21–30^ papers were selected for inclusion in this review (figure 1).

### Study characteristics

Table 1 provides an overview of the study design and study population of all 10 studies. All the studies were observational studies of RA cohorts; none contained patients with IP. The majority of studies were set in the USA^21^ ^23^ ^25^ ^28–30^ and were typical RA populations with respect to age and gender except for the study by Cannon *et al*,^28^ who utilised data from the Veterans Affairs Rheumatoid Arthritis (VARA) registry. Information about MTX dosage, either the starting dose or average dose, was present in five studies,^21^ ^26–28^ ^30^ and only four studies reported an average dose.^21^ ^26–28^

**Table 1:** Studies investigating adherence to MTX treatment

Study	Place	N	Study design	RA definition	Age (years)	Percentage of females	Disease severity	Disease duration (years)	First time or established MTX user	Follow-up	Average MTX dose mg/week
Harley *et al*^21^	USA	2662	Retro	ICD9	53.3±14.7	73	NP	NP	First time	365 days	10±NP
de Klerk *et al*^22^	NED	127	Prosp cohort	Rheum	60±14*	66*	NP	NP	First time	210 days	NP
Grijalva *et al*^23^	USA	14 932	Retro cohort	ICD9	54 (44–63)	78	NP	NP	First time	NP	NP
Contreras-Yanez *et al*^24^	MEX	93	Prosp cohort	Rheum	40.8±13.9†	80†	DAS28: 2.1±1.1†	<1†	Established	6 months	NP
Grijalva *et al*^25^	USA	14 586	Retro cohort	ICD9	55 (45–64)†	76†	NP	NP	First time	NP	NP
de Thurah *et al*^26^	DEN	941	Retro cohort	ICD10	60.5	69	Erosions 71%	52.9%<5	First time	384 (233–931) days	15
de Thurah *et al*^27^	DEN	103	Prosp cohort	ICD10	63 (32–80)	64	Erosions 60%	6.3 (0–27)	First time	9 months	13.8 (12.5–15.1)
Cannon *et al*^28^	USA	455	Retro cohort	ACR 1987	64±11	8	DAS: 3.9±1.6	NP	First time	42.7±3 1.2 months	16±4
Salt and Frazier^29^	USA	108	Cross-sectional	ACR 1987	52±13†	76†	NP	10±10†	Established	NA	NP
Waimann *et al*^30^	USA	111	Prosp cohort	ACR 1987	NP	87†	DAS28: 4.7±1.6†	8±6†	Both	24 months	NP

Median (IQR), mean (95%CI) otherwise mean±SD.

†Values represent total RA sample and not specific to MTX users.

ACR1987, American College of Rheumatology Classification 1987 Criteria; DAS, Disease Activity Score; DEN, Denmark; ICD, International Statistical Classification of Diseases; MEX, Mexico; MTX, methotrexate; NED, Netherlands; NP, information not presented; Prosp, prospective; RA, rheumatoid arthritis; Retro, retrospective; Rheum, RA diagnosed by a rheumatologist.

### Quality of the included studies

Overall, the quality of the studies was considered high in 7/10 studies;^21^ ^23^ ^25–28^ ^30^ all 10 studies used reproducible methods of adherence measurement, 8/10 studies used sampling methods that reduced bias^21^ ^23–28^ ^30^ and 7/10 studies had a response rate of more than 80%.^21^ ^23–28^ Where associations with other factors were tested, 7/8 studies presented statistics with CIs^23^ 25–30 and 7/8 studies used analysis that accounted for the skewed adherence data.22–24 27–30 See online supplementary table S3 for a full description.

### Rates of adherence to MTX

Rates of adherence differed in how they were calculated and presented between studies. Adherence was reported as a proportion or percentage in six,^22–26^ ^30^ and participants were categorised as non-adherent and adherent in four.^21^ ^26^ ^28^ ^29^ In general, estimates of adherence to MTX ranged from 59% to 107% (table 2).

**Table 2:** Comparison of MTX rates of adherence across studies

Study	QA score	Quality rating	Adherence definition	Adherence is primary outcome?	Data on predictors of adherence?	Data on adherence and outcomes?	N	MTX adherence rate	95% CI/SD
*MEMS*									
de Klerk *et al*^22^	12	Low	Per cent of adherence (ratio)	Yes	Yes	No	23	107%	98 to 117
Waimann *et al*^30^	15	High	Per cent of adherence (ratio)	Yes	Yes	Yes	76	63%	20%
*Pharmacy refill*									
Harley *et al*^21^	8	High	Per cent of adherent (MPR ≥80%)	Yes	No	No	1668	64%	24 to 102
Grijalva *et al*^23^	15	High	Per cent of adherence (MPR)	Yes	Yes	No	2933	80%	NP
Grijalva *et al*^25^	9	High	Per cent of adherence (MPR)	No	No	No	NP	59%	31 to 82
de Thurah *et al*^26^	14	High	Per cent of non-adherence (CMG)	Yes	Yes	No	941	12%	1113
Cannon *et al*^28^	15	High	Per cent of adherent (MPR ≥80%)	Yes	No	Yes	384	84%	NP
*Self-report*									
Contreras-Yanez *et al*^24^	11	Low	Per cent of adherent (7-day DRR ≥80%)	Yes	Yes	Yes	10	78%	NP
de Thurah *et al*^27^	14	High	Per cent of non-adherent (CQ-R ≤25th centile)	Yes	Yes	No	85 65	BL 23% 9 mo 23%	NP NP
Salt and Frazier^29^	9	Low	Per cent of adherent (MARS ≥39)	Yes	Yes	No	77	92%	NP

9 mo, 9 months; BL, baseline; CMG, continuous medication gap; CQ-R, Compliance Questionnaire-Rheumatology; DRR, Drug Record Registry; MARS, Medication Adherence Revised Scale; MEMS, Medication Event Monitoring System; MPR, medication possession ratio; MTX, methotrexate; NP, information not presented.

Across studies various methods were used to determine adherence. Two studies^22^ ^30^ used Medication Electronic Monitoring Systems (MEMS), which captures details of pill bottle openings, and is considered an accurate indirect method to evaluate adherence.^31^ Waimann *et al*^30^ reported that the average percentage of correctly taken doses was 63% (SD 20%), with underdosing accounting for 22% (SD 18%) of non-adherence and overdosing 14% (SD 10%). A study judged to be of low quality used the same methodology^22^ and reported 107% (95% CI 98% to 117%) average adherence representing overdosage.

Five studies determined adherence according to pharmacy refill records,^21^ ^23^ ^25^ ^26^ ^28^ two of which categorised optimal adherence as ≥80% and the proportion of adherent patients was 63.7% and 84%.^21^ ^28^ Two studies conceptualised adherence as a dimension and raw medication possession ratios (MPR) were reported, ranging from 59% to 80%.^23^ ^25^ One study reported gaps in medication possession, using this method non-adherence was estimated to be 12%.^26^

Where studies used self-report methods, rates of adherence ranged from 78% to 92%.^24^ ^29^ One study reported 23% of patients were non-adherent based on questionnaires scores.^27^ Two out of the three self-report studies did not define the recall period.^27^ ^29^ Salt and Frazier^29^ used the validated Medication Adherence Revised Scale (MARS), which required patients to endorse the frequency from ‘not at all’ to ‘very often’ they engaged in specific non-adherent behaviours, for example, altering the dose, taking a drug holiday or forgetting to take their medication.^32^ de Thurah *et al*^27^ used the Compliance Questionnaire-Rheumatology (CQ-R), a validated self-report measure that consists of 19 items that do not ask directly about MTX use, instead patients endorsed the extent to which they held adherent attitudes.^33^ Contreras-Yanez *et al*^24^ used a 7-day diary to record the day, timing and dose of MTX over a 7-day period before a clinic appointment at three time points at two monthly intervals. Adherence over 6 months was calculated by dividing the reported MTX use at the three time points by the expected MTX use. Patients were categorised as adherent if they took 80% or more of MTX as prescribed.

None of the selected studies directly measured adherence to MTX but Contreras-Yanez *et al*^24^ measured MTX concentrations in serum to evaluate MTX persistence. Whereas 100% of participants reported MTX persistence using a ‘7-day diary’, serum-detected persistence was lower, indicated by the moderate agreement (κ=0.67, p<0.0001) between diary-recorded persistence and serum concentration of MTX, although this result is difficult to interpret in light of variable timing of blood tests in relation to MTX dosing.^34^

### Factors associated with adherence

Seven studies investigated 38 factors and their association with adherence to MTX in RA.^22–24^ ^26^ ^27^ ^29^ ^30^

#### Demographic factors

Six studies investigated demographic factors (table 3).^22^ ^24^ ^26^ ^27^ ^29^ ^30^ Overall there was weak evidence that demographic factors were associated with adherence. de Klerk *et al*^22^ reported being female improved adherence, but this was not replicated in four other studies.^24^ ^26^ ^27^ ^29^ de Thurah *et al*^26^ reported being older than 67 years was associated with non-adherence, but four other studies found no association with age.^24^ ^27^ ^29^ ^30^ Salt and Frazier^29^ revealed that ethnicity (white vs non-white) in the unadjusted analysis was strongly associated with adherence; however, this was not replicated by Waimann *et al*.^30^ The latter study found being married, and living with someone was associated with better adherence but this was a univariate association unadjusted for other factors.

**Table 3:** Summary of evidence for demographic predictors of adherence to MTX

Predictor	Study	N	Outcome	Unadjusted ES (95% CI)/univariate analyses	p Value	Adjusted ES (95% CI)	p Value
*Gender*							
Per cent of male	Contreras-Yanez *et al*^24^*	93	Adherent (CQ ≥9) vs non-adherent (CQ ≤8)	8 (17%) vs 5 (11.9%)	0.55	NP	NP
	Salt and Frazier^29^*	108	Adherent (MARS ≥39) vs non-adherent (MARS ≤38)	23 (24%) vs 4 (32%)	0.69	NP	NP
Male vs female	de Thurah *et al*^26^	941	Non-adherence (CMG)	12.0 (10.5 to 13.5) vs 12.5 (11.4 to 13.5)	NS		
Being male	de Thurah *et al*^27^†	85	Non-adherence (CQR ≤25th centile) at BL	PR 0.7 (0.2 to 1.7)	NS	PR 0.8 (0.3 to 2.0)	NS
	de Thurah *et al*^27^†	65	Non-adherence (CQR ≤25th centile) at 9 mo	PR 0.4 (0.1 to 1.3)	NS	PR 0.3 (0.1 to 1.3)	NS
	de Klerk *et al*^22^*,‡	127	Per cent of adherence (MEMS)	β 13.5 (NP)	<0.05	0.38 (NP)	<0.05
*Age*							
Age in years	Salt and Frazier^29^*	108	Adherent (MARS ≥39) vs non-adherent (MARS ≤38)	52±14 vs 53±9	0.77	OR 1.01 (0.94 to 1.08)	0.8
	Contreras-Yanez *et al*^24^*	93	Adherent (CQ ≥9) vs non-adherent (CQ ≤8)	42.7±14.1 vs 38.9±13.4	0.18	NP	NP
	Waimann *et al*^30^	107	Per cent of adherence (MEMS)	r −0.07	>0.20	NP	NP
>67 vs <55 years old	de Thurah *et al*^26^	941	Non-adherence (CMG)	13.1 (11.6 to 14.6) vs 10.8 (9.3 to 12.3)	<0.01	Not defined	Sig
>55	de Thurah *et al*^27^†	85	Non-adherence (CQR ≤25th centile) at BL	0.5 (0.2 to 1.1)	NS	PR 0.6 (0.2 to 1.6)	NS
>55	de Thurah *et al*^27^†	65	Non-adherence (CQR ≤25th centile) at 9 mo	PR 0.9 (0.4 to 2.0)	NS	PR 0.7 (0.3 to 1.7)	NS
*Ethnicity*							
White vs non-white	Salt and Frazier^29^*	108	Adherent (MARS ≥39) vs non-adherent (MARS ≤38)	84 (86%) vs 5 (56%)	0.04	OR 10.1 (1.66 to 61.4)	0.01
Hispanic vs white vs African-American	Waimann *et al*^30^	107	Per cent of adherence (MEMS)	66±17 vs 64±20 vs 60±24	NS	NP	NP
*Being single/living alone*							
Single	Contreras-Yanez *et al*^24^*	93	Adherent (CQ ≥9) vs non-adherent (CQ ≤8)	23 (49%) vs 25 (54%)	0.68	NP	NP
	Salt and Frazier^29^*	108	Adherent (MARS ≥39) vs non-adherent (MARS ≤38)	16 (16%) vs 2 (22%)	0.74	OR 1.44 (0.15 to 13.7)	0.75
Widowed/separated vs married	Waimann *et al*^30^	107	Per cent of adherence (MEMS)	56±19 vs 72±16	<0.01	NP	NP
Widowed/separated vs single	Waimann *et al*^30^	107	Per cent of adherence (MEMS)	56±19 vs 69±18	<0.01	NP	NP
Living alone vs not living alone	Waimann *et al*^30^	107	Per cent of adherence (MEMS)	56±21 vs 66±19	<0.05	NP	NP
*Education*							
< High school vs ≥ high school	Waimann *et al*^30^	107	Per cent of adherence (MEMS)	67±19 vs 62±20	NS	NP	NP
Years of education	Contreras-Yanez *et al*^24^*	93	Adherent (CQ ≥9) vs non-adherent (CQ ≤8)	10.1±4.2 vs 11.4±3.5	0.09	NP	NP
	Salt and Frazier^29^*	108	Non-adherence (MARS ≤38)	OR 1.09 (1.50 to 0.79)	0.61	NP	NP
School >10 years	de Thurah *et al*^27^†	85	Non-adherence (CQR ≤25th centile) at BL	PR 1.7 (0.7 to 3.8)	NS	PR 1.5 (0.5 to 4.1)	NS
	de Thurah *et al*^27^†	65	Non-adherence (CQR ≤25th centile) at 9 mo	PR 1.3 (0.6 to 3.1)	NS	PR 1.0 (0.3 to 2.8)	NS
*Residence (rural vs urban)*	Salt and Frazier^29^*	108	Adherent (MARS ≥39) vs non-adherent (MARS ≤38)	55 (51%) vs 48 (53%)	0.18	OR 7.52 (0.70 to 83.3)	0.1
*Employment status*							
Employed vs unemployed	Contreras-Yanez *et al*^24^*	93	Adherent (CQ ≥9) vs non-adherent (CQ ≤8)	16 (34%) vs 15 (33%)	1	NP	NP
Employed	Salt and Frazier^29^*	108	Adherent (MARS ≥39) vs non-adherent (MARS ≤38)	27 (26%) vs 25 (26%)	0.72	OR 2.19 (21.3 to 0.21)	0.52
Employed vs unemployed	Waimann *et al*^30^	104	Per cent of adherence (MEMS)	64±15 vs 63±14	NS	NP	NP
*Income <$20 000/year vs* ≥*$20 000/year*	Waimann *et al*^30^	90	Per cent of adherence (MEMS)	62±19 vs 69±20	NS	NP	NP
*Low socioeconomic status*	Contreras-Yanez *et al*^24^*	93	Adherent (CQ ≥9) vs non-adherent (CQ ≤8)	43 (92%) vs 40 (87%)	0.52	NP	NP
*Uninsured vs private vs medicaid*	Waimann *et al*^30^	103	Per cent of adherence (MEMS)	65±15 vs 66±13 vs 64±19	NS	NP	NP
*English vs Spanish language*	Waimann *et al*^30^	107	Per cent of adherence (MEMS)	64±21 vs 65±19	NS	NP	NP

*Studies judged low quality.

†MTX adherence.

‡Includes RA, PMR and gout.

9 mo, 9 months; BL, baseline; CMG, continuous measure of medication gaps; CQ, Compliance Questionnaire; ES, effect size; MEMS, Medication Electronic Monitoring System; CQR, Compliance Questionnaire-Rheumatology; MARS, Medication Adherence Revised Scale; MTX, methotrexate; NP, information not presented; NS, non-significant; PR, prevalence ratio; r, Pearson correlation coefficient; RA, rheumatoid arthritis; Sig, significant; β, regression coefficient.

#### Psychological factors

Only three studies tested psychological factors (see table 4),^22^ ^27^ ^30^ but psychological factors consistently associated with adherence. de Thurah *et al*^27^ found higher levels of baseline adherence in patients with high beliefs about the necessity of MTX; however, this association only remained at 9 months in the unadjusted analysis. In comparison, low concerns about MTX were not associated with higher adherence at baseline or at 9 months, although there was a trend for MTX concerns to become more predictive over time. In unadjusted analyses, Waimann *et al*^30^ demonstrated that good mental health indicated by lower scores on the Center for Epidemiological Studies Depression Scale 10-item survey (CES-D10), and higher scores on the mental component summary of the Medical Outcomes Study Questionnaire (MOS SF-12 MCS), were significantly associated with lower adherence rates. de Klerk *et al*^22^ examined several psychological predictors; non-avoidant coping, passive reactive coping and self-efficacy with regard to taking medications significantly associated with higher adherence. Further, de Klerk *et al* found that patient reported lower quality of life as measured by the European Quality of Life measure (EuroQol) and the Nottingham Health Profile (NHP) were associated with lower adherence. This finding was not replicated by Waimann *et al*^30^ where health-related quality of life was measured using the physical component summary of the Medical Outcomes Study Questionnaire (MOS SF-12 PCS).

**Table 4:** Summary of evidence for disease-related and psychological predictors of adherence to MTX

Predictor	Study	N	Adherence outcome	Unadjusted effect size (95% CI)/univariate analyses	p Value	Adjusted effect size (95% CI)	p Value
*RA duration*							
Years	Salt and Frazier^29^*	108	Non-adherent (MARS ≤38)	NP	NP	OR 1.00 (1.01 to 1.00)	0.83
	Waimann *et al*^30^	107	Adherence (MEMS)	r 0.08	>0.20	NP	NP
1–5	de Thurah *et al*^26^†	941	Non-adherence (CMG)	β 0.03 (0.01 to 0.06)	Sig	β 0.01 (−0.01 to 0.04)	NS
>5	de Thurah *et al*^26^†	941	Non-adherence (CMG)	β 0.02 (−0.01 to 0.04)	NS	β −0.04 (−0.07 to −0.02)	Sig
>5	de Thurah *et al*^27^†	85	Non-adherence (CQR ≤25th centile) at BL	PR 1.7 (0.7 to 4.1)	NS	PR 1.5 (0.5 to 4.7)	NS
>5	de Thurah *et al*^27^†	65	Non-adherence (CQR ≤25th centile) at 9 mo	PR 1.5 (0.6 to 3.6)	NS	PR 1.2 (0.4 to 3.1)	NS
*Inflammatory biomarkers*							
CRP	Contreras-Yanez *et al*^24^*	93	Adherent (CQ ≥9) vs non-adherent (CQ ≤8)	2.4±2.6 vs 2.6±2.4	0.68	NP	NP
CRP 8–32	de Thurah *et al*^26^†	941	Non-adherence (CMG)	β 0.00 (−0.02 to 0.02)	NS	β −0.02 (−0.04 to 0.01)	NS
CRP >32	de Thurah *et al*^26^†	941	Non-adherence (CMG)	β −0.02 (−0.05 to 0.01)	NS	β −0.04 (−0.07 to −0.02)	Sig
Erythrocyte sedimentation rate	Contreras-Yanez *et al*^24^*	93	Adherent (CQ ≥9) vs non-adherent (CQ ≤8)	24.1±17.4 37.5±23.8	0.003	NP	NP
*Disease Activity Score-28*							
	Contreras-Yanez *et al*^24^*	93	Adherent (CQ ≥9) vs non-adherent (CQ ≤8)	3.6±1.3 vs 5.1±1.9	≤0.001	NP	NP
	Waimann *et al*^30^	90	Per cent of adherent (MEMS)	r −0.27	0.01	NP	NP
*Sharp score*	Waimann *et al*^30^	79	Per cent of adherent (MEMS)	r −0.06	>0.20	NP	NP
*Functional ability*							
HAQ	de Klerk *et al*^22^*,‡	127	Per cent of adherence (MEMS)	ANOVA (no data)	NS	NP	NP
	Contreras-Yanez *et al*^24^*	93	Adherent (CQ ≥9) vs non-adherent (CQ ≤8)	0.2±0.4 vs 0.4±0.5	0.04	NP	NP
HAQ >1.75	de Thurah *et al*^27^†	85	Nonadherence (CQR ≤25th centile)	PR 1.2 (0.5 to 2.5)	NS	PR 1.4 (0.6 to 3.1)	NS
0.75–1.75	de Thurah *et al*^27^†	65	Non-adherence (CQR ≤25th centile) at BL	PR 1.5 (0.5 to 4.9)	NS	PR 0.8 (0.2 to 3.3)	NS
HAQ >1.75	de Thurah *et al*^27^†	65	Non-adherence (CQR ≤25th centile) at 9 mo	PR 0.8 (0.3 to 2.5)	NS	PR 1.0 (0.2 to 3.4)	NS
Modified—HAQ	Waimann *et al*^30^	107	Per cent of adherence (MEMS)	r −0.20	0.04	NP	NP
*Comorbidity*							
Number of comorbidities	Waimann *et al*^30^	107	Per cent of adherence (MEMS)	r −0.06	>0.20	NP	NP
Per cent with comorbidity	Contreras-Yanez *et al* 24*	93	Adherent (CQ ≥9) vs non-adherent (CQ ≤8)	40% (85) vs 36% (78.3)	0.43	NP	NP
Any vs none	de Thurah *et al*^27^†	85	Non-adherence (CQR ≤25th centile) at BL	PR 1.3 (0.4 to 3.9)	NS	PR 1.1 (0.4 to 3.3)	NS
	de Thurah *et al*^27^†	65	Non-adherence (CQR ≤25th centile) at 9 mo	PR 1.3 (0.4 to 3.8)	NS	PR 2.2 (0.5 to 9.7)	NS
COPD	de Thurah *et al*^26^†	941	Non-adherence (CMG)	β 0.00 (−0.04 to 0.04)	NS	β 0.04 (0.00 to 0.07)	Sig
Diabetes	de Thurah *et al*^26^†	941	Non-adherence (CMG)	β −0.04 (−0.1 to 0.02)	NS	β 0.00 (−0.05 to 0.05)	NS
Liver disease	de Thurah *et al*^26^†	941	Non-adherence (CMG)	β 0.06 (0.02 to 0.10)	Sig	β 0.04 (0.00 to 0.08)	Sig
*BMQ low concern about MTX*	de Thurah *et al*^27^†	85	Non-adherence (CQR ≤25th centile) at BL	PR 0.8 (0.4 to 1.8)	NS	PR 0.7 (0.3 to 1.8)	NS
	de Thurah *et al*^27^†	65	Non-adherence (CQR ≤25th centile) at 9 mo	PR 0.5 (0.2 to 1.2)	NS	PR 0.5 (0.2 to 1.3)	NS
*BMQ high perceptions of MTX necessity*	de Thurah *et al*^27^†	85	Non-adherence (CQR ≤25th centile) at BL	PR 0.4 (0.1 to 0.8)	Sig	PR 0.3 (0.1 to 0.8)	Sig
	de Thurah *et al*^27^†	65	Non-adherence (CQR ≤25th centile) at 9 mo	PR 0.2 (0.1 to 0.6)	Sig	PR 0.4 (0.1 to 1.1)	NS
*LTMBS (self-efficacy)*	de Klerk *et al*^22^‡	127	Per cent of adherence (MEMS)	F 5.9	0.02	NP	NP
UCL avoidant coping	de Klerk *et al*^22^*,‡	127	Per cent of adherence (MEMS)	NP	NS	β −0.41	< 0.05
*Passive reactive coping*							
UCL passive coping	de Klerk *et al*^22^*,‡	127	Per cent of adherence (MEMS)	NP	NS	β 0.79	<0.05
UCL reactive coping	de Klerk *et al*^22^*,‡	127	Per cent of adherence (MEMS)	NP	NS	β 0.4	<0.05
UCL active coping	de Klerk *et al*^22^*,‡	127	Per cent of adherence (MEMS)	NP	NS	NP	NP
UCL reassuring thoughts	de Klerk *et al*^22^*,‡	125	Per cent of adherence (MEMS)	NP	NS	NP	NP
*Mental health*							
CES-D10	Waimann *et al*^30^	107	Per cent of adherence (MEMS)	r −0.19	0.05	NP	NP
MOS SF-12 MCS	Waimann *et al*^30^	107	Per cent of adherence (MEMS)	r 0.34	<0.01	NP	NP
MOS social support	Waimann *et al*^30^	107	Per cent of adherence (MEMS)	r 0.17	0.08	NP	NP
*Health-related quality of life*							
European Quality of Life Measure	de Klerk *et al*^22^*,‡	127	Per cent of adherence (MEMS)	F 5.42	<0.01	NP	NP
RA Quality of Life Measure	de Klerk *et al*^22^*,‡	81	Per cent of adherence (MEMS)	F 0.21	0.65	NP	NP
Nottingham Health Profile	de Klerk *et al*^22^*,‡	127	Per cent of adherence (MEMS)	NP	NS	β −0.62	<0.05
MOS SF-12 Physical Component Summary	Waimann *et al*^30^	107	Per cent of adherence (MEMS)	r 0.07	>0.20	NP	NP

*Studies judged low quality.

†MTX adherence.

‡Includes RA, PMR and gout.

9 mo, 9 months; ANOVA, analysis of variance; BL, baseline; BMQ, Beliefs in Medicines Questionnaire; CES-D10, Centre of Epidemiologic Studies Depression Scale; CMG, continuous medication gap; COPD, chronic obstructive pulmonary disease; CQ, Compliance Questionnaire; CQR, Compliance Questionnaire—Rheumatology; CRP, C reactive protein; F, ANOVA test statistic; HAQ, Health Assessment Questionnaire; MARS, Medication Adherence Revised Scale; MEMS, Medicine Event Monitoring System; MOS, Medical Outcomes Study; MTX, methotrexate; NP, not presented; NS, non-significant; PR, prevalence ratio; r, Pearson correlation coefficient; RA, rheumatoid arthritis; SF-12 MCS, Mood Component Summary of MOS 12-item Short Form Health Survey; Sig, significant; UCL, Utrecht Coping List; β, regression coefficient.

#### Disease-related factors

Six studies investigated disease-related factors (table 4).^22^ ^24^ ^26^ ^27^ ^29^ ^30^ One study suggested adherence reduced with increasing disease duration,^26^ but this finding was not replicated in three other studies.^27^ ^29^ ^30^ Two studies measured disease activity using DAS28,^24^ ^30^ and reported higher DAS28 score to be associated with lower adherence. In one study, there was no observed association between the inflammatory erythrocyte sedimentation rate and a negative association between C reactive protein (CRP) and adherence,^24^ whereas, in another study, high CRP was associated with increased adherence.^26^ In unadjusted analyses, two studies found that disability was associated with lower adherence rates,^24^ ^30^ but two other studies did not replicate these findings.^22^

#### Treatment-related factors

Five studies investigated treatment-related factors (table 5).^23^ ^24^ ^26^ ^27^ ^30^ Grijalva *et al*^23^ found adherence to MTX monotherapy was higher compared with MTX in combination with another sDMARD or biological DMARD (bDMARD). Contreraz-Yanez *et al*^24^ reported a similar trend; however, only MTX in combination with three other DMARDs reached statistical significance. In contrast, Waimann *et al*^30^ found the addition of a bDMARD, or number of RA-related drugs did not affect adherence to MTX. One study found no effect of MTX dose or folic acid use on adherence,^27^ and one study reported no association between incidence of adverse events (AEs) and adherence.^22^

**Table 5:** Summary of evidence for treatment-related predictors of adherence to MTX

Predictor	Study	N	Outcome	Unadjusted ES (95% CI)/univariate analyses	p Value	Adjusted ES (95% CI)	p Value
*MTX dose*							
MTX 12.5–17.5 mg	de Thurah *et al*^27^*	85	Non-adherence (CQR ≤25th centile) at BL	PR 0.6 (0.2 to 1.5)	NS	PR 0.7 (0.3 to 1.7)	NS
>17.5 mg/week	de Thurah *et al*^27^*	85	Non-adherence (CQR ≤25th centile) at BL	PR 0.5 (0.2 to 1.7)	NS	PR 0.6 (0.1 to 2.4)	NS
MTX 12.5–17.5 mg	de Thurah *et al*^27^*	65	Non-adherence (CQR ≤25th centile) at 9 mo	PR 0.2 (0.0 to 1.7)	NS	PR 0.4 (0.0 to 3.9)	NS
>17.5 mg/week	de Thurah *et al*^27^*	65	Non-adherence (CQR ≤25th centile) at 9 mo	PR 1.0 (0.4 to 2.4)	NS	PR 1.1 (0.4 to 3.1)	NS
*Other sDMARDs*							
MTX + HCQ vs MTX	Grijalva *et al*^23^	NP	Adherence (MPR)	β 0.13 (0.14 to 0.11)	NP	β 0.11 (0.13 to 0.09)	<0.001
MTX + HCQ vs MTX	Contreras-Yanez *et al*^24^†	93	≥80% adherent (7-day DRR)	OR 3.9 (0.64 to 23.05)	0.14	NP	NP
MTX + HCQ + SSZ + LEF vs MTX	Contreras-Yanez *et al*^24^†	70	≥80% adherent (7-day DRR)	OR 21 (1.5 to 293)	0.02	NP	NP
MTX + HCQ + SSZ vs MTX	Contreras-Yanez *et al*^24^†	70	≥80% adherent (7-day DRR)	OR 3.7 (0.68 to 20.2)	0.13	NP	NP
MTX + SSZ vs MTX	Contreras-Yanez *et al*^24^†	70	≥80% adherent (7-day DRR)	OR 5.3 (0.49 to 56.8)	0.17	NP	NP
*Other bDMARDs*							
MTX + INF vs MTX	Grijalva *et al*^23^	NP	Adherence (MPR)	β 0.12 (0.07 to 0.18)	NP	β 0.12 (0.07 to 0.17)	<0.001
MTX + ETA vs MTX	Grijalva *et al*^23^	NP	Adherence (MPR)	β 0.12 (0.09 to 0.15)	NP	β 0.11 (0.08 to 0.14)	<0.001
MTX + ADA vs MTX	Grijalva *et al*^23^	NP	Adherence (MPR)	β 0.06 (0.02 to 0.10)	NP	β 0.07 (0.03 to 0.11)	0.001
Biological yes vs no	Waimann *et al*^30^	107	Per cent of adherence (MEMS)	63±21 vs 66±17	NS	NP	NP
	Salt and Frazier^29^†	108	Non-adherent (MARS ≤38)	NP	NP	OR 1.26 (0.63 to 2.53)	0.51
*Drugs related to RA*							
Number of RA drugs	Contreras-Yanez *et al*^24^†	93	Adherent (CQ ≥9) vs non-adherent (CQ ≤8)	4.8±1.5 vs 4.7±1.4	0.65	NP	NP
	Waimann *et al*^30^	107	Per cent of adherence (MEMS)	r 0.05	>0.20	NP	NP
Pills/day of RA drugs	Waimann *et al*^30^	107	Per cent of adherence (MEMS)	r 0.08	>0.20	NP	NP
*Drugs unrelated to RA*							
Number of drugs for comorbidity	Contreras-Yanez *et al*^24^†	93	Adherent (CQ ≥9) vs non-adherent (CQ ≤8)	3.2±1.7 vs 3.2±1.9	0.94	NP	NP
Number of non-RA drugs	Waimann *et al*^30^	107	Per cent of adherence (MEMS)	r −0.17	0.07	NP	NP
Pills/day of non-RA drugs	Waimann *et al*^30^	107	Per cent of adherence (MEMS)	r −0.15	0.12	NP	NP
*Adverse events*	de Klerk *et al*^22^†,‡	127	Per cent of adherence (MEMS)	NP	NS	NP	NP
*Folic acid use*	de Thurah *et al*^27^*	85	Non-adherence (CQR ≤25th centile) at BL	PR 0.2 (0.0 to 1.6)	NS	PR 0.3 (0.0 to 2.7)	NS
	de Thurah *et al*^27^*	65	Non-adherence (CQR ≤25th centile) at 9 mo	PR 0.6 (0.2 to 2.4)	NS	PR 0.4 (0.1 to 2.6)	NS

*MTX adherence.

†Studies judged low quality.

‡Includes RA, PMR and gout.

9 mo, 9 months; ADA, adalimumab; bDMARD, biological disease-modifying antirheumatic drug; BL, baseline; CQ, Compliance Questionnaire; CQR, Compliance Questionnaire—Rheumatology; DRR, drug record registry; ES, effect size; ETA, etanercept; HCQ, hydroxychloroquine; INF, infliximab; LEF, leflunomide; MARS, Medication Adherence Revised Scale; MEMS, Medicine Event Monitoring System; MPR, medication possession ratio; MTX, methotrexate; NP, not presented; NS, non-significant; PR, prevalence ratio; r, Pearson correlation coefficient; RA, rheumatoid arthritis; sDMARD, synthetic disease-modifying antirheumatic drug; Sig, significant; SSZ, sulfasalazine; β, regression coefficient.

### Associations with patient-reported and clinical outcomes

Only a few studies investigated the association between adherence and clinical outcomes (n=3),^24^ ^28^ ^30^ patient-reported outcomes (n=2),^28^ ^30^ and radiographic damage (n=1).^30^ Despite study heterogeneity, all three studies observed a negative association between adherence and treatment response. One study investigated adherence to MTX alone^28^ with the other two studies including other DMARDs within the analysis.

Contreras-Yanez *et al*^24^ reported that self-reported non-adherent patients who were in remission at baseline were more at risk of a disease flare than adherent patients during follow-up (48.41 per 100 person/years vs 13.31 per 100 person/years, p<0.002), the relative risk of non-adherence was borderline significant when adjusted for other factors (RR=4.8 (0.8 to 27.6), p=0.08).

The main finding of Cannon *et al*^28^ was that being adherent (MPR ≥80%) negatively associated with change in DAS28 over follow-up in unadjusted and adjusted analyses for the entire cohort (β=−0.34 (−0.68 to −0.06), p<0.05), adjusted (β=−0.37 (−0.67 to −0.07), p<0.05). A subanalysis compared the effect of adherence on outcomes for established and first-time users of MTX. There was a significant negative association between being adherent and DAS28 response in the established user cohort (β=−0.38 (−0.67 to −0.05) p<0.05, β_adj_=−0.37 (−0.72 to −0.02), p<0.05), but this negative association did not reach significance in the first-time user cohort (β=−0.54 (−1.18 to 0.11), p>0.05, β_adj_=−0.40 (−1.11 to 0.30), p>0.05).^28^

Waimann *et al*^30^ reported a small negative association between adherence (MEMS) and disease activity when adjusted for other factors (β=−0.2 p=0.03). Non-adherent patients (MEMS ≤80%) had consistently greater radiographic damage than adherent patients did at baseline (58 vs 80, p=0.01) and by 12 months (61 vs 86, p=0.02), but this difference lost significance at 24 months (69 vs 87, p=0.12).^30^ See online supplementary table S4 for a description of all the associations between adherence to MTX and patient outcomes.

## Discussion

This systematic review found some evidence that adherence to MTX is suboptimal. In this review, mean adherence could be summarised as suboptimal (59–63%) in two studies,^25^ ^30^ optimal in two studies (80–88%),^23^ ^26^ and in one study mean adherence was 107% indicating MTX non-adherence through overuse.^22^ Three studies dichotomised patients into adherent and non-adherent groups based on indirect measurement of MTX doses taken and the percentage of patients who had optimal adherence ranged from 64% to 85%.^21^ ^24^ ^28^ Two studies defined patients as adherent based on questionnaire scores, and the proportion classed adherent ranged from 77% to 92%.^27^ ^29^ The variation observed in this review probably reflects differences in definition and measurement of adherence, sample characteristics and size, study design and statistical models. This heterogeneity meant that it was not possible to perform a combined meta-analysis to produce an overall estimate of adherence or the factors influencing it.

Evidence synthesis revealed a high prevalence of psychological factors that impacted MTX adherence. Higher baseline DAS28 was associated with reduced adherence in two studies suggesting that patients with more severe baseline disease activity have reduced adherence to MTX.^24^ ^30^ All three studies that examined the impact of MTX non-adherence on clinical outcomes reported that suboptimal adherence was significantly associated with reduced response to treatment.^24^ ^28^ ^30^

All indirect measures of adherence have limited validity due to the assumptions one has to make. One has to assume the self-reported behaviour on a questionnaire, or affiliated behaviour of bottle opening, or prescription collection, is equivalent to actually taking the medication.^31^ The generalisability of findings in this review obtained using these methods were constrained by the well-understood issues of small sample sizes^22^ ^24^ and using cohorts obtained from US Medical insurance companies^21^ ^23^ ^25^ and the US Veteran registry.^28^

Two studies^27^ ^29^ used questionnaires to assess adherence to MTX that have been validated for use with RA populations.^32^ ^33^ However, the psychometric properties of the MARS and the CQ-R have been tested in a RA population and shown to be multidimensional,^29^ which suggests they may be measures of important correlates of adherence, rather than adherence per se.

A limitation that applied to all the existing studies was a failure to detect medically advised missed doses. Patients can be advised to lower or miss doses when they experience AEs; therefore, adherence rates may be underestimated. de Thurah *et al*^26^ performed a sensitivity analysis that excluded weeks where antibiotics were co-prescribed from the calculation of MPR, and reported MPR increased slightly; however, there are several other valid reasons for a person with RA to temporarily halt MTX. For example, in the same study, ulcer/mild liver disease was negatively associated with MTX adherence.^26^ It is feasible that this was due to the association of MTX with abnormalities of liver function and thus did not represent true non-adherence.

Taking the above limitations into account we concluded the strongest evidence was for psychological predictors of adherence, such as treatment beliefs, coping styles and mood.^22^ ^27^ ^30^ Unfortunately, there was little cross-over between studies with respect to the measures used to assess beliefs, coping and mood to make strong recommendations for specific predictors. Some studies were cross-sectional,^27^ limiting the establishment of a causal relationship between psychological factors and adherence. In studies of other diseases patients with higher perceived necessity of MTX assessed the long-term benefits of MTX use more positively, and placed a higher emphasis on good adherence;^35^ this supports the findings of de Thurah *et al*.^27^ A high perception of need for medication is influenced by previous experiences, expectations of the disease and therapy, and current symptoms.^36^ Therefore, perceived need can be expected to change over time and patients with early RA may perceive less necessity compared with patients with established RA.^37^ Increasing perceived necessity has previously been suggested as an intervention to improve adherence in other diseases, as has psychological therapy such as cognitive behaviour therapy and motivational psychology.^38^

The finding that more severe disease was associated with reduced adherence contradicts a recent systematic review that synthesised data for chronic and acute diseases. The authors reported that the severity of chronic diseases, which included RA, correlated with improved adherence.^39^ One possible explanation for the finding from the current review is that prior non-adherence before the start of study might have contributed to more severe disease at baseline. However, the association between disease severity and non-adherence may also indicate the mediating role of particular illness beliefs that are triggered by disease events and lead to decisions to non-adhere. Alternatively, patients with more active disease may have received higher doses of MTX, and experienced more AEs, which led to reduced adherence due to increased concerns about the medication.^40^

### Limitations of review

This was a thorough review of 10 papers pertaining to MTX adherence in a RA population but there were some limitations. First, the review was limited to English articles; however, this was unlikely to change the overall findings or recommendations for future research as we only excluded nine abstracts on that basis. Second, the QA tool used was bespoke to the current review and not a validated measure; however, the domains of the tool were based on a systematic review of quality measures for observational studies.^19^ Further, the QA tool is presented to guide the reader, and not to exclude articles deemed low quality.

We searched for articles which studied inflammatory arthritis and RA populations in order to reflect current clinical practice; sometimes patients start MTX who clinically look like RA but do not strictly fulfil classification criteria for RA. Our search did not retrieve any early disease cohorts where the classification criteria for RA had not been fulfilled, but we did include two papers^22^ ^24^ where the classification criteria had not been applied. In these studies, we do not know if all the patients would have been classified as having RA, but we considered it important to include these papers in the final results.

### Research recommendations

*Measuring adherence*: This review has highlighted gaps in knowledge with respect to MTX adherence; first, research is needed that addresses the extent to which patients are genuine non-adherers or are adhering to medical advice and not taking MTX; second, the reasons for MTX non-adherence are not known and the predictors of intentional non-adherence are likely to be different to those who unintentionally forget to take MTX; third, studies should be designed to include multiple measures of adherence to compensate for the inherent limitations of each methodology.

#### Sample selection

An important unobserved confounder of any association between a potential predictor and MTX non-adherence is prior non-adherent behaviour. Therefore, samples need to exclude patients who have used MTX before. Retrospective studies have to make assumptions when defining cohorts as first-time users; therefore, prospective inception cohort studies are needed to overcome this problem.

#### Investigation of psychological predictors

The causal role of psychological factors in determining adherence to MTX needs to be addressed urgently. The extent to which patient beliefs, coping styles and mood can be said to predict adherence can only be addressed in specifically designed prospective cohort studies that rigorously assess modifiable illness and treatment beliefs over time. The reviewed studies tended to examine psychological, disease or treatment-related predictors in isolation, and further studies are required to investigate the possible interplay between psychology, treatment and illness in determining non-adherence.

## Conclusion

In conclusion, this systematic review shows adherence to MTX does impact patient clinical outcomes, and therefore it is important to address. Estimates of adherence vary widely; currently, there is no direct test for MTX adherence; further research is therefore required to develop a direct reliable test of adherence. This review highlights a number of modifiable patient factors including treatment beliefs, self efficacy around medicine taking and coping styles that were shown to associate with MTX adherence; these factors require further research and may lead to interventions that will improve MTX adherence.

## References

[1] Maetzel A, Wong A, Strand VS Meta-analysis of treatment termination rates among rheumatoid arthritis patients receiving disease-modifying anti-rheumatic drugs. Rheumatology (Oxford) 2000;39:975–81. 10.1093/rheumatology/39.9.975 10986302

[2] Smolen JS, Landewe R, Breedveld FC EULAR recommendations for the management of rheumatoid arthritis with synthetic and biological disease-modifying antirheumatic drugs: 2013 update. Ann Rheum Dis 2014;73:492–509. 10.1136/annrheumdis-2013-204573 24161836 PMC3933074

[3] National Institute for Health and Care Excellence. Rheumatoid arthritis: the management of rheumatoid arthritis in adults. CG79. Manchester: NICE, 2009.

[4] Emery P, Breedveld FC, Hall S Comparison of methotrexate monotherapy with a combination of methotrexate and etanercept in active, early, moderate to severe rheumatoid arthritis (COMET): a randomised, double-blind, parallel treatment trial. Lancet 2008;372:375–82. 10.1016/S0140-6736(08)61000-4 18635256

[5] Emery P, Burmester GR, Bykerk VP Evaluating drug-free remission with abatacept in early rheumatoid arthritis: results from the phase 3b, multicentre, randomised, active-controlled AVERT study of 24 months, with a 12-month, double-blind treatment period. Ann Rheum Dis 2015;74:19–26. 10.1136/annrheumdis-2014-206106 25367713 PMC4283672

[6] Hider SL, Silman A, Bunn D Comparing the long-term clinical outcome of treatment with methotrexate or sulfasalazine prescribed as the first disease-modifying antirheumatic drug in patients with inflammatory polyarthritis. Ann Rheum Dis 2006;65:1449–55. 10.1136/ard.2005.049775 16540547 PMC1798363

[7] The World Health Organisation. Adherence to long-term therapies: evidence for action. 2003 http://www.who.int/chp/knowledge/publications/adherence_full_report.pdf?ua=1 (accessed March 2013).

[8] Trueman P, Taylor DG, Lowson K Evaluation of the scale, causes and costs of waste medicines. Report of DH funded national project YHEC/The School of Pharmacy, University of London 2010.

[9] Cramer J, Rosenheck R, Kirk G VA Naltrexone Study Group. Medication compliance feedback and monitoring in a clinical trial: predictors and outcomes. Value Health 2003;6:566–73. 10.1046/j.1524-4733.2003.65269.x 14627063

[10] Haynes RB, McDonald HP, Garg AX Helping patients follow prescribed treatment: clinical applications. JAMA 2002;288:2880–3. 10.1001/jama.288.22.2880 12472330

[11] Jackevicius CA, Mamdani M, Tu JV Adherence with statin therapy in elderly patients with and without acute coronary syndromes. JAMA 2002;288:462–7. 10.1001/jama.288.4.462 12132976

[12] Pasma A, van't Spijker A, Hazes JM Factors associated with adherence to pharmaceutical treatment for rheumatoid arthritis patients: a systematic review. Semin Arthritis Rheum 2013;43:18–28. 10.1016/j.semarthrit.2012.12.001 23352247

[13] Fidder HH, Singendonk MM, van der Have M Low rates of adherence for tumor necrosis factor-alpha inhibitors in Crohn's disease and rheumatoid arthritis: results of a systematic review. World J Gastroenterol 2013;19:4344–50. 10.3748/wjg.v19.i27.4344 23885145 PMC3718902

[14] Blum MA, Koo D, Doshi JA Measurement and rates of persistence with and adherence to biologics for rheumatoid arthritis: a systematic review. Clin Ther 2011;33:901–13. 10.1016/j.clinthera.2011.06.001 21715007

[15] de Achaval SD, Suarez-Almazor ME Treatment adherence to disease-modifying antirheumatic drugs in patients with rheumatoid arthritis and systemic lupus erythematosus. Int J Clin Rheumtol 2010;5:313–26. 10.2217/ijr.10.15 20676388 PMC2910438

[16] Koncz T, Pentek M, Brodszky VS Adherence to biologic DMARD therapies in rheumatoid arthritis. Expert Opin Biol Ther 2010;10:1367–78. 10.1517/14712598.2010.510508 20681888

[17] Harrold LR, Andrade SE Medication adherence of patients with selected rheumatic conditions: a systematic review of the literature. Semin Arthritis Rheum 2009;38:396–402. 10.1016/j.semarthrit.2008.01.011 18336875 PMC2695137

[18] da Costa Santos CM, de Mattos Pimenta CA, Nobre MR The PICO strategy for the research question construction and evidence search. Rev Lat Am Enfermagem 2007;15:508–11. 10.1590/S0104-11692007000300023 17653438

[19] Sanderson S, Tatt ID, Higgins JP Tools for assessing quality and susceptibility to bias in observational studies in epidemiology: a systematic review and annotated bibliography. Int J Epidemiol 2007;36:666–76. 10.1093/ije/dym018 17470488

[20] van Tulder M, Furlan A, Bombardier C Updated method guidelines for systematic reviews in the Cochrane collaboration back review group. Spine 2003;28:1290–9. 10.1097/01.BRS.0000065484.95996.AF 12811274

[21] Harley CR, Frytak JR, Tandon N Treatment compliance and dosage administration among rheumatoid arthritis patients receiving infliximab, etanercept, or methotrexate. Am J Manag Care 2003;9(6 Suppl):S136–43. 14577718

[22] de Klerk E, van der Heijde D, Landewe R Patient compliance in rheumatoid arthritis, polymyalgia rheumatica, and gout. J Rheumatol 2003;30:44–54. 12508389

[23] Grijalva CG, Chung CP, Arbogast PG Assessment of adherence to and persistence on disease-modifying antirheumatic drugs (DMARDs) in patients with rheumatoid arthritis. Med Care 2007;45(Suppl 2):S66–76. 10.1097/MLR.0b013e318041384c 17909386

[24] Contreras-Yanez I, Ponce de Leon S, Cabiedes J Inadequate therapy behavior is associated to disease flares in patients with rheumatoid arthritis who have achieved remission with disease-modifying antirheumatic drugs. Am J Med Sci 2010;340:282–90. 10.1097/MAJ.0b013e3181e8bcb0 20881757

[25] Grijalva CG, Kaltenbach L, Arbogast PG Adherence to disease-modifying antirheumatic drugs and the effects of exposure misclassification on the risk of hospital admission. Arthritis Care Res (Hoboken) 2010;62:730–4. 10.1002/acr.20087 20191470 PMC2945370

[26] de Thurah A, Norgaard M, Johansen MB Methotrexate compliance among patients with rheumatoid arthritis: the influence of disease activity, disease duration, and co-morbidity in a 10-year longitudinal study. Scand J Rheumatol 2010;39:197–205. 10.3109/03009740903251318 20085505

[27] de Thurah A, Norgaard M, Harder I Compliance with methotrexate treatment in patients with rheumatoid arthritis: influence of patients’ beliefs about the medicine. A prospective cohort study. Rheumatol Int 2010;30:1441–8. 10.1007/s00296-009-1160-8 19823840

[28] Cannon GW, Mikuls TR, Hayden CL Merging Veterans Affairs rheumatoid arthritis registry and pharmacy data to assess methotrexate adherence and disease activity in clinical practice. Arthritis Care Res (Hoboken) 2011;63:1680–90. 10.1002/acr.20629 21905260 PMC5497696

[29] Salt E, Frazier SK Predictors of medication adherence in patients with rheumatoid arthritis. Drug Dev Res 2011;72:756–63. 10.1002/ddr.20484 22267889 PMC3261653

[30] Waimann CA, Marengo MF, de Achaval S Electronic monitoring of oral therapies in ethnically diverse and economically disadvantaged patients with rheumatoid arthritis: consequences of low adherence. Arthritis Rheum 2013;65:1421–9. 10.1002/art.37917 23728826 PMC3691007

[31] Quittner AL, Modi AC, Lemanek KL Evidence-based assessment of adherence to medical treatments in pediatric psychology. J Pediatr Psychol 2008;33:916–36. 10.1093/jpepsy/jsm064 17846042 PMC2639495

[32] Horne R, Weinman J Patients’ beliefs about prescribed medicines and their role in adherence to treatment in chronic physical illness. J Psychosom Res 1999;47:555–67. 10.1016/S0022-3999(99)00057-4 10661603

[33] de Klerk E, van der Heijde D, Landewe R The compliance-questionnaire-rheumatology compared with electronic medication event monitoring: a validation study. J Rheumatol 2003;30:2469–75. 14677194

[34] Godfrey C, Sweeney K, Miller K The population pharmacokinetics of long-term methotrexate in rheumatoid arthritis. Br J Clin Pharmacol 1998;46:369–76. 10.1046/j.1365-2125.1998.t01-1-00790.x 9803986 PMC1874158

[35] Miller W, Rollnick S Motivational interviewing. New York: Guilford Press, 1999.

[36] Horne R Patients’ beliefs about treatment: the hidden determinant of treatment outcome? J Psychosom Res 1999;47:491–5. 10.1016/S0022-3999(99)00058-6 10661596

[37] Neame R, Hammond A Beliefs about medications: a questionnaire survey of people with rheumatoid arthritis. Rheumatology (Oxford) 2005;44:762–7. 10.1093/rheumatology/keh587 15741193

[38] Haynes RB, Ackloo E, Sahota N Interventions for enhancing medication adherence. Cochrane Database Syst Rev 2008;(2):CD000011 10.1002/14651858.CD000011.pub3 18425859

[39] DiMatteo MR, Haskard KB, Williams SL Health beliefs, disease severity, and patient adherence: a meta-analysis. Med Care 2007;45:521–8. 10.1097/MLR.0b013e318032937e 17515779

[40] Hoekstra M, van Ede AE, Haagsma CJ Factors associated with toxicity, final dose, and efficacy of methotrexate in patients with rheumatoid arthritis. Ann Rheum Dis 2003;62:423–6. 10.1136/ard.62.5.423 12695153 PMC1754533

